# Cationic BODIPY Photosensitizers for Mitochondrion-Targeted Fluorescence Cell-Imaging and Photodynamic Therapy

**DOI:** 10.3390/pharmaceutics15051512

**Published:** 2023-05-16

**Authors:** Isabel Wen Badon, Jun-Pil Jee, Temmy Pegarro Vales, Chanwoo Kim, Seungbin Lee, Jaesung Yang, Si Kyung Yang, Ho-Joong Kim

**Affiliations:** 1 Department of Chemistry, Chosun University, Gwangju 61452, Republic of Korea; istbadon@gmail.com; 2 Department of Life Sciences, Chung-Ang University, Seoul 06974, Republic of Korea; 3 Drug Delivery Research Lab, College of Pharmacy, Chosun University, Gwangju 61452, Republic of Korea; jee@chosun.ac.kr; 4 Department of Chemistry, Caraga State University, Butuan City 8600, Philippines; valestemmy@gmail.com; 5 Mineral Resources Management Research and Training Center, Caraga State University, Butuan City 8600, Philippines; 6 Department of Chemistry, Yonsei University, Wonju 26493, Republic of Korea; chanwoo.kim@yonsei.ac.kr (C.K.); dltmdqls0503@gmail.com (S.L.); 7 Department of Chemistry Education, Chonnam National University, Gwangju 61186, Republic of Korea

**Keywords:** BODIPY, cationic, mitochondria, photodynamic therapy, photosensitizer

## Abstract

The straightforward synthesis of three cationic boron-dipyrromethene (BODIPY) derivatives and their mitochondria-targeting and photodynamic therapeutic (PDT) capabilities are reported. Two cancer cell lines (HeLa and MCF-7) were used to investigate the PDT activity of the dyes. Compared to their non-halogenated counterparts, halogenated BODIPY dyes exhibit lower fluorescence quantum yields and enable the efficient production of singlet oxygen species. Following LED light irradiation at 520 nm, the synthesized dyes displayed good PDT capabilities against the treated cancer cell lines, with low cytotoxicity in the dark. In addition, functionalization of the BODIPY backbone with a cationic ammonium moiety enhanced the hydrophilicity of the synthesized dyes and, consequently, their uptake by the cells. The results presented here collectively demonstrate the potential of cationic BODIPY-based dyes as therapeutic drugs for anticancer photodynamic therapy.

## 1. Introduction

Photodynamic therapy (PDT) is an innovative, minimally invasive and controlled modality of treating disease that employs a photosensitizer (PS), light, and oxygen (^3^O_2_). The mode of treatment involves the generation of singlet oxygen (^1^O_2_) and other reactive oxygen species (ROS) from localized PS to induce tissue destruction in areas where these three crucial components merge. Many PS agents have been employed in PDT, including noble metal complexes [1,2], organic framework compounds [3,4], and organic fluorophores (e.g., porphyrins [5], phthalocyanines [6], indocyanine [7], and boron-dipyrromethene (BODIPY) [8]). Nevertheless, several drawbacks, including structural instability, poor photostability, and low light-to-dark toxicity ratios, prevent the clinical application of the vast majority of PS compounds. Moreover, many typical PS agents are produced using complex synthetic processes and are soluble only in selected solvents. Thus, there is a growing need to synthesize an innovative class of photosensitizers for PDT that are stable, highly efficient, simple to synthesize, and suitable for a wide range of conditions.

Since its discovery, BODIPY has been established as a versatile fluorophore. BODIPY-based fluorophores have demonstrated immense potential as PDT agents [9,10,11]. These dyes are at the forefront of basic and medical research because of their highly desirable spectral features, including resistance to photobleaching, high fluorescence quantum yield, high extinction coefficients, and higher light-to-dark toxicity ratios relative to those of other PS agents. In addition, BODIPY dyes can be easily synthesized and post-synthetically modified to tune their photophysical attributes and ROS production. Over the past ten years, scientific literature has described a vast array of synthetic strategies and structures [12,13,14,15,16,17]. For instance, BODIPYs for photodynamic therapy are typically incorporated with heavy atoms and/or transition metals to induce an efficient intersystem crossing (ISC) of molecules from the singlet (S_1_) to the triplet (T_1_) state. Additionally, the configuration and presence of rotatable bonds can induce a phototoxic effect [18,19]. The molecule in the triplet state then collides with nearby molecular oxygen simultaneously with energy transfer, elevating the molecular oxygen to its singlet state. Singlet oxygen (^1^O_2_) is cytotoxic to cancer cells. Photodynamic therapy combined with diagnosis using fluorescence imaging is the future of advanced theragnostic applications [20]. Hence, photosensitizers that generate ^1^O_2_ while also functioning as fluorescent probes are in high demand.

Cellular localization plays a critical function in determining the success of PSs in PDT applications. Owing to its highly hydrophobic core, BODIPY lacks selectivity for cancer cells. Thus, it is necessary to functionalize BODIPY analogs to improve their effectiveness in targeting cancer cells while retaining their PDT efficiency and excellent bioimaging capability. Several mitochondria-targeting probes typically employ lipophilic and cationic moieties, including organic phosphine salts [21], mitochondria-targeting peptides [22], and quaternary ammonium salts [23]. Moreover, incorporating cations in BODIPY dyes not only imparts hydrophilicity but also affords organelle-targeting ability. The targeting effect of these molecules depends on the negative membrane potential of the inner mitochondrial membrane, which leads to their accumulation in the mitochondrial matrix through an inverse concentration gradient [21]. Herein, the cationic moiety is generated by the quaternization of the dimethylamine group at the *meso* position. Cationic BODIPY with an *N,N,N*-trimethylamino group at the *meso* position has been shown to produce ^1^O_2_; however, it needs to interact with micelles to reduce vibrational decay in order to show a significant photodynamic effect [24]. In addition, halogenation at positions two and six of the BODIPY core is a recognized strategy for imparting photodynamic activity via the heavy-atom effect [25]. Moreover, as far as the authors know, this is the first report on the use of diaminophenyl-functionalized BODIPY and its quaternized form in cell imaging and photodynamic therapy. Nonetheless, BODIPY dyes have also been used as optical pH sensors. The results described herein fill the gap in the functionality of this type of BODIPY dye.

## 2. Materials and Methods

### 2.1. Materials

All chemicals were acquired commercially. Boron trifluoride diethyl etherate (BF_3_·Et_2_O), trifluoroacetic acid (TFA), 4-(Dimethylamine)benzaldehyde, and 2,4-Dimethylpyrrole were procured from Sigma Aldrich (St. Louis, MO, USA). *N*-Bromosuccinimide (NBS) and *N*-iodosuccinimide (NIS) were obtained from TCI Chemicals (Tokyo, Japan). 2,3-Dichloro-5,6-dicyano-1,4-benzoquinone (DDQ) was sourced from Alfa Aesar (Haverhill, MA, USA). *N*,*N*-Diethylethanamine (TEA), iodomethane, sodium hydrogen carbonate (NaHCO_3_), and magnesium sulfate (MgSO_4_) were obtained from Daejung Chemical (Gyeonggi-do, Republic of Korea). Analytical grade solvents were used and distilled before the experiments as needed.

### 2.2. Synthesis of Cationic BODIPY Dyes

#### 2.2.1. Acid-Catalyzed Condensation Reaction (BODIPY H1)

The same synthetic route based on our previous publication was followed to obtain the BODIPY dye **H1** [26]. 4-(Dimethylamine)benzaldehyde (0.58 g, 4.73 mmol) and 2, 4-Dimethylpyrrole (0.9 g, 9.46 mmol) were dissolved in dried CH_2_Cl_2_ (120 mL) under Argon gas at room temperature. This was followed by the immediate addition of a catalytic amount of TFA. After stirring overnight, DDQ (1.07 g, 4.73 mmol) was dissolved in distilled CH_2_Cl_2_ and injected dropwise into the solution. After 2 h, TEA (4 mL) was slowly injected into the mixture, followed by the addition of BF_3_·Et_2_O (4.5 mL) in a similar manner. The reaction was quenched after 1.5 h, and the crude product was extracted with CH_2_Cl_2_. Then, the organic fraction was dried with MgSO_4_, filtered, and concentrated until dry. BODIPY **H1** was isolated by silica gel column chromatography as a yellow-red solid.

**H1**: (Yield: 260.1 mg, 18.2%) ^1^H NMR (300 MHz, CDCl_3_): δ = 7.07 (d, 2H, *J* = 9 Hz,2Ar-H), 6.79 (d, 2H, *J* = 9 Hz, 2Ar-H), 5.97 (s, 2H, 2Ar-H), 3.02 (s, 6H, 2CH_3_), 2.55 (s, 6H, 2CH_3_), and 1.48 (s, 6H, 2CH_3_) ppm.

#### 2.2.2. Halogenation Reactions (BODIPY Br1 and I1)

The C-2 and C-6 positions of the BODIPY dye **H1** were halogenated using either NBS or NIS. Briefly, 1.0 eq of H1 was dissolved in distilled CH_2_Cl_2_ at room temperature. Thereafter, 3.0 eq of either NBS or NIS was added to the reaction. When the reaction was finished, the solvent was evaporated, and the halogenated **Br1** and **I1** products were isolated using column chromatography with silica gel, respectively.

**Br1**: (Yield: 79.7 mg, 55.8%) ^1^H NMR (300 MHz, CDCl_3_): δ = 7.04 (d, 2H, *J* = 9 Hz, 2Ar-H), 6.80 (d, 2H, *J* = 9 Hz, 2Ar-H), 3.04 (s, 6H, 2CH_3_), 2.60 (s, 6H, 2CH_3_), and 1.49 (s, 6H, 2CH_3_) ppm.

**I1**: (Yield: 137.5 mg, 81.6%) ^1^H NMR (300 MHz, CDCl_3_): δ = 7.04 (d, 2H, *J* = 9 Hz, 2Ar-H), 6.80 (d, 2H, *J* = 9 Hz, 2Ar-H), 3.05; (s, 6H, 2CH_3_), 2.64 (s, 6H, 2CH_3_), and 1.50 (s, 6H, 2CH_3_) ppm.

#### 2.2.3. Methylation Reaction (AmH, AmBr and AmI)

Methylation reactions of the dimethylamine moiety at the *meso* position were carried out by dissolving BODIPY precursors (**H1**, **Br1**, and **I1**, respectively) with 2 mL iodomethane in 5 mL anhydrous acetonitrile. The reaction was kept stirring at room temperature for two days before being purified by column chromatography using aluminum oxide.

**AmH**: (Yield: 50.0 mg, 60.0%) ^1^H NMR (300 MHz, CD_3_OD): δ = 8.15 (d, 2H, *J* = 9 Hz, 2Ar-H), 7.69 (d, 2H, *J* = 9 Hz, 2Ar-H), 6.11 (s, 2H, 2Ar-H), 3.76 (s, 9H, 3CH_3_), 2.50 (s, 6H, 2CH_3_), 1.41 (s, 6H, 2CH_3_) ppm. ^13^C NMR (300 MHz, CD_3_OD): δ = 157.48, 149.39, 144.25, 140.43, 138.62, 131.82, 122.71, 122.51, 57.87, and 14.86 ppm. HRMS (ESI): *m*/*z* 382.2267, calculated mass for C_22_H_27_BF_2_IN_3_ 382.28

**AmBr**: (Yield: 80.0 mg, 79.0%) ^1^H NMR (300 MHz, CD_3_OD): δ = 8.30 (d, 2H, *J* = 9 Hz, 2Ar-H), 7.60 (d, 2H, *J* = 9 Hz, 2Ar-H), 4.11 (s, 9H, 3CH_3_), 2.61 (s, 6H, 2CH_3_), 1.33 (s, 6H, 2CH_3_) ppm. ^13^C NMR (300 MHz, CD_3_OD): δ = 154.92, 140.13, 132.80, 131.79, 121.93, 112.45, 57.98, 44.38, 27.33, and 14.08 ppm. HRMS (ESI): *m*/*z* 540.0468, calculated mass for C_22_H_25_BBr_2_F_2_IN_3_ 540.07.

**AmI**: (Yield: 120.0 mg, 71.0%) ^1^H NMR (300 MHz, CD_3_OD): δ = 8.16 (d, 2H, *J* = 9 Hz, 2Ar-H), 7.67 (d, 2H, *J* = 9 Hz, 2Ar-H), 3.74 (s, 9H, 3CH_3_), 2.54 (s, 6H, 2CH_3_), 1.36 (s, 6H, 2CH_3_) ppm. ^13^C NMR (300 MHz, CD_3_OD): δ = 138.47, 131.88, 122.92, 113.71, 57.94, 40.48, 27.28, 17.71, 16.46, and 16.28 ppm. HRMS (ESI): *m*/*z* 634.0198, calculated mass for C_22_H_25_BF_2_I_3_N_3_ 634.07.

### 2.3. Spectroscopic Measurements

#### 2.3.1. Structural Characterization

The structures of BODIPY dyes were confirmed by ^1^H and ^13^C NMR spectroscopy using a Bruker AM250 spectrometer (Billerica, MA, USA) at 300 MHz frequency with TMS as a reference at 25 °C. The high-resolution electrospray ionization mass spectrometry (HR-ESI-MS) data of the dyes was measured using SYNAPT G2-Si high-definition mass spectrometer (Wilmslow, UK).

#### 2.3.2. Spectroscopic and Photophysical Properties

The steady-state absorption spectra of the dyes were measured at room temperature on a spectrophotometer (LAMBDA 25; PerkinElmer, Waltham, MA, USA) and a fluorometer (LS-55; PerkinElmer, Waltham, MA, USA), respectively. For the measurements, the sample solution was contained in a quartz cuvette with an optical path length of 1 cm. The fluorescence quantum yield (Φ_F_) was calculated by a comparative method based on the following equation:

(1) $$\Phi_{F}^{S}=\Phi_{F}^{R}\left(\frac{A_{R}}{A_{S}}\right)\left(\frac{E_{S}}{E_{R}}\right){\left(\frac{n_{S}}{n_{R}}\right)}^{2}$$

where *A* stands for the optical density at the excitation wavelength, *E* refers to the integrated fluorescence intensity, and *n* is the refractive index of the solvent used. The superscripts R and S represent reference and sample, respectively. Rhodamine 6G (Φ_F_ = 0.95 in ethanol) was used as a reference.

#### 2.3.3. Singlet Oxygen Generation Efficiency

The singlet oxygen quantum yield (Φ_Δ_) was measured by the photosensitized oxidation of DPBF, which is a ^1^O_2_ quencher. A methanolic solution containing DPBF (A_410nm_~1.0) and Am*X* dyes was prepared. The optical density of each BODIPY dye was adjusted to be A_500nm_~0.1 for AmH and A_522nm_~0.1 for AmBr and AmI. The solution was exposed to a green LED light (λ_max_ = 500 nm or 522 nm) with an intensity of 7 mW cm^−2^ and a beam diameter of 0.7 cm. The absorption spectrum was obtained periodically between 0–10 min to monitor the attenuation of the absorption band at 410 nm as a result of the photooxidation of DPBF. After assuming the first-order kinetics, the Φ_Δ_ was determined based on the following equation:

(2) $$\Phi_{∆}^{S}=\Phi_{∆}^{R}\left(\frac{m_{S}}{m_{R}}\right)\left(\frac{F_{R}}{F_{S}}\right)$$

where *m* means the slope of the time-dependent absorbance attenuation curve and *F* (*F* = 1–10−^A^) is the factor that corrects for the difference in absorbance between reference and sample. The reference used was Eosin Y (EY, Φ_Δ_ = 0.26 in methanol).

### 2.4. Cells and Culture Conditions

HeLa and MCF-7 cancer cell lines were supplied by the Korean Cell Line Bank. The cell lines were kept in RPMI 1640 medium (Gibco, Carlsbad, CA, USA) containing 10% heat-inactivated fetal bovine serum (FBS). Penicillin (100 U/mL) and streptomycin (100 mg/mL) were also added to the media as antibiotics (WELGENE Inc., Gyeongsangbuk-do, Republic of Korea). The cell lines were kept at 37 °C and a 5% CO_2_ environment.

### 2.5. Assessment of Cell Proliferation

To evaluate the response of cell lines towards the BODIPY dyes, the colorimetric-based CellTiter 96^®®^ AQueous One Solution Cell Proliferation Assay (Promega, Madison, WI, USA) was used. In 96-well plates, HeLa and MCF-7 cells were seeded at a density of 2 × 10^3^ cells/well and incubated at 37 °C and 5% CO_2_ for 1 day. Varying doses (0–1600 nM) of BODIPY dyes were applied to the cells for 1 day. Next, the CellTiter 96^®®^ AQueous One Solution Reagent was pipetted into each well of the culture plate, followed by 4-hr incubation. Finally, the absorbance of the solution was measured at 490 nm with an ELISA plate reader (ThermoFisher Scientific, Inc., Waltham, MA, USA).

### 2.6. Photodynamic Activity Assay

To assess the cell proliferation of the cancer cell lines with photodynamic treatment, the same aforementioned colorimetric-based assay was used. HeLa and MCF-7 cells were cultured in similar conditions with an extended 2-h incubation at 37 °C and 5% CO_2_ in the dark. Next, the media were changed with phenol-red free RPMI 1640 and irradiated for 10 min with a green light-emitting diode (LED, 520 nm). The cells were incubated for 24 h. Then, the CellTiter 96^®®^ AQueous One Solution Reagent was added into each well of the culture plate, followed by 4-hr incubation. The absorbance of the solution was measured at 490 nm using an ELISA plate reader (ThermoFisher Scientific, Inc., Waltham, MA, USA).

### 2.7. Fluorescence Cell Imaging

HeLa and MCF-7 cells were administered with 1.6 μM BODIPY dyes for 24 h and further incubated for 45 min with the mitochondria-stain MitoTracker Red (Invitrogen, Waltham, MA, USA). The cells were fixed with 4% paraformaldehyde for 10 min, permeabilized with 0.1% Triton X-100 for 10 min, and then treated with 4,6-diamidino-2-phenylindole (DAPI) as a counterstain for 1 h at room temperature. The fluorescent cells were imaged using the Zeiss LSM-700 confocal microscope (Zeiss, Oberkochen, Germany).

### 2.8. Statistical Analysis

Assays were conducted in triplicates (*n* = 3), and data are presented as means ± standard deviations of the means. Group mean variations were analyzed using Tukey’s test and 1-way analysis of variance (ANOVA) on GraphPad Prism 6 software (San Diego, CA, USA). The averages of the groups were considered significantly different when *p* < 0.05.

### 2.9. Theoretical Calculations

Geometry optimization and electronic structure calculations were performed using the density functional theory (DFT) and time-dependent DFT (TD-DFT) with the B3LYP functional and the 6-31G(d) basis sets of the Gaussian 16 program package. All DFT and TD-DFT computations were performed using water as solvent. The spin-orbit coupling (SOC) matrix elements were calculated at the B3LYP/ZORA-def2-TZVP level of theory using the ORCA 5.0.1 [27,28] program.

## 3. Results

### 3.1. Synthesis of Cationic BODIPY Derivatives

**Scheme 1:**
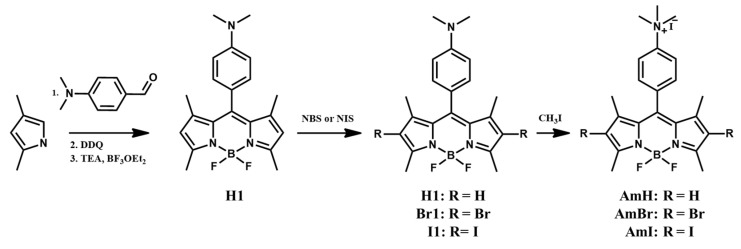
Synthetic scheme of the cationic BODIPY dyes **AmH**, **AmBr**, and **AmI**.

A schematic of the synthesis of cationic BODIPY is shown in Scheme 1. Initially, a trifluoroacetic acid-catalyzed reaction between 2,4-dimethylpyrrole and 4-dimethylaminobenzaldehyde was performed to produce a highly unstable intermediate, the dipyrromethene hydrochloride salt. Subsequent oxidation by DDQ and complexation in situ with BF_3_·Et_2_O produced the BODIPY core **H1**. In the second step, heavy bromine and iodine atoms were introduced into the 2,6-positions of the BODIPY H1 by employing NBS and NIS as the bromine and iodine sources, respectively. It is well known that the introduction of heavy atoms into BODIPY and aza-BODIPY analogs considerably boosts the spin-orbit coupling of different states. This improves the intersystem crossing efficiency and, as a result, enhances the singlet oxygen quantum yield. By modifying the proportional amounts of succinimides used and the reaction time, the halogenated BODIPY dyes **Br1** and **I1** were obtained in high yields of 56% and 82%, respectively. The last step quaternizes the dimethylamino moiety of the BODIPY dyes using iodomethane, a common methylation reagent, via a simple SN2 nucleophilic substitution reaction. Three cationic BODIPY derivatives, **AmH**, **AmBr**, and **AmI**, were produced via facile methylation at room temperature in high yields (60%, 79%, and 71%, respectively). In contrast, the previously reported methylation route of **AmH** uses DMF as a solvent and involves heating the reaction at 40 C for 72 hrs [24]. ^1^H-NMR spectroscopy was used to characterize all the synthesized BODIPY dyes, whereas additional ^13^C-NMR and mass spectroscopic characterizations were done on the cationic BODIPY photosensitizers, **AmH**, **AmBr**, and **AmI** (Figures S1–S12 in the Supplementary Material).

### 3.2. Photophysical Properties and Computational Analysis of Cationic BODIPY Dyes

**Figure 1:**
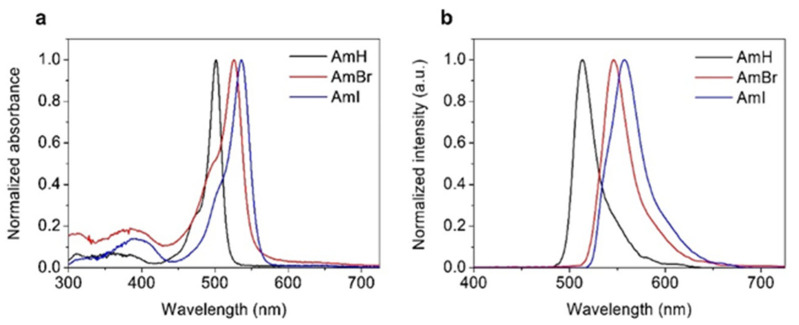
(**a**) Absorption and (**b**) emission spectra of cationic BODIPY dyes in methanol (steady-state). The respective absorption maxima of the solutions were used as excitation wavelengths.

The photophysical features of the cationic BODIPY derivatives **AmH**, **AmBr**, and **AmI** were measured using UV/Vis absorption and fluorescence. Table 1 summarizes the key absorption and emission parameters, and Figure 1 shows the absorbance and emission spectra of the synthesized cationic BODIPY dyes in methanolic solution; these spectra are similar to those of previously characterized BODIPY dyes [29,30,31]. The synthesized cationic BODIPYs exhibited the typical absorption and emission profiles of conventional BODIPY dyes (Figure 1a): a distinctly tapered absorption band with peaks in the range of 501–536 nm, assigned to the characteristic strong S_0_ → S_1_ transition of the BODIPY chromophore [32], and a second, significantly weaker, broad absorption band attributable to the S_0_ → S_2_ transition on the short-wavelength side of the spectra [33]. The synthesized dyes also showed BODIPY emission spectra (Figure 1b), which were narrow and mirror images of their absorption spectra, evidencing the homology of the molecular structures of the dyes.

Notably, the absorption and emission wavelengths of the halogenated BODIPY dyes were red-shifted. This translates into significant bathochromic shifts in the absorption (25–35 nm) and emission (33–45 nm) maxima relative to their non-halogenated counterparts. Moreover, the iodinated BODIPY derivative demonstrated a more dramatic red-shift in its absorption maximum than the brominated counterpart. This was expected because iodine is heavier than bromine and would provide a more noticeable heavy-atom effect on the resulting BODIPY dyes [34]. The fluorescence quantum yields (Φ_F_) of the BODIPY-based dyes were measured against the rhodamine 6G standard (Φ_F_ = 0.95). The fluorescence quantum yields of **AmH**, **AmBr**, and **AmI** are shown in Table 1.

**Figure 2:**
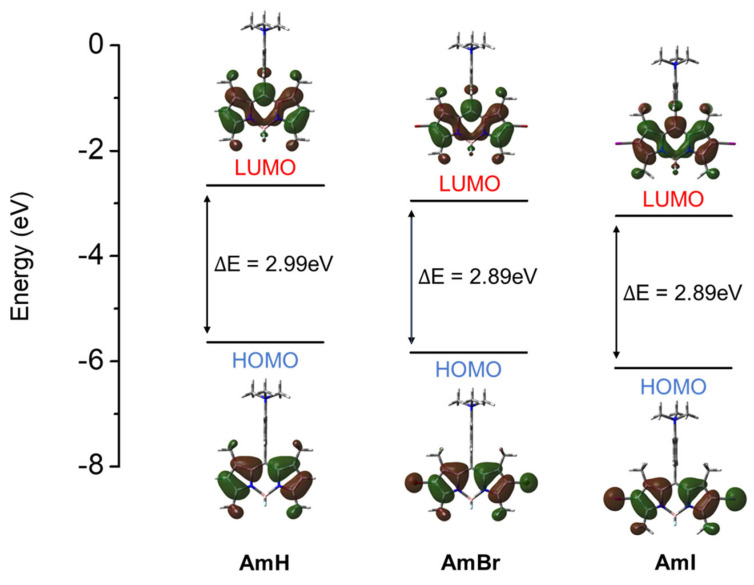
The frontier molecular orbitals and their respective energy levels of cationic BODIPY dyes. The calculations were performed under a water solvent using B3LYP functional and 6-31G(d) basis sets for **AmH** and **AmBr** and 6-31G(d,p) basis set (LanL2DZ basis for **AmI**).

The significant reduction in the reported ΦF for halogenated BODIPY dyes implies that due to the heavy-atom effect, ISC from a singlet to a triplet excited state, which is a prerequisite process for singlet oxygen generation in triplet PSs, occurred more efficiently in the halogenated BODIPY dyes **AmBr** and **AmI** than in the non-halogenated **AmH**. Furthermore, the optimized molecular structures of the BODIPY compounds were utilized to investigate the frontier molecular orbitals (MOs) for further understanding of the electronic effects of halogenation on the BODIPY properties. The calculated photophysical properties are shown in Table S1 (SI). Although the theoretical absorption maxima and spectral shifts were found to be less than the experimental results for all compounds, approximately 70–76 nm less than the experimental results), the qualitative trend was well reproduced by the calculations. The halogenated BODIPYs **AmBr** and **AmI** displayed a calculated redshift of 31–41 nm compared to the experimental redshift of 25–35 nm. Moreover, upon comparing the frontier orbitals, the narrowing of the highest occupied molecular orbital-lowest unoccupied molecular orbital (HOMO-LUMO) gap, as seen in Figure 2, was largely caused by a destabilization of the HOMO induced by the halogens, resulting in enhanced oscillator strength and a red-shift of the absorption band. This was confirmed by the computed oscillator strength and contribution of the frontier orbitals to each transition, as summarized in Table S1. The obtained theoretical and experimental photophysical data indicated that the synthesized cationic BODIPY dyes have the potential for fluorescence cell imaging and photodynamic therapy.

### 3.3. Singlet Oxygen Generation of Cationic BODIPY Derivatives

**Figure 3:**
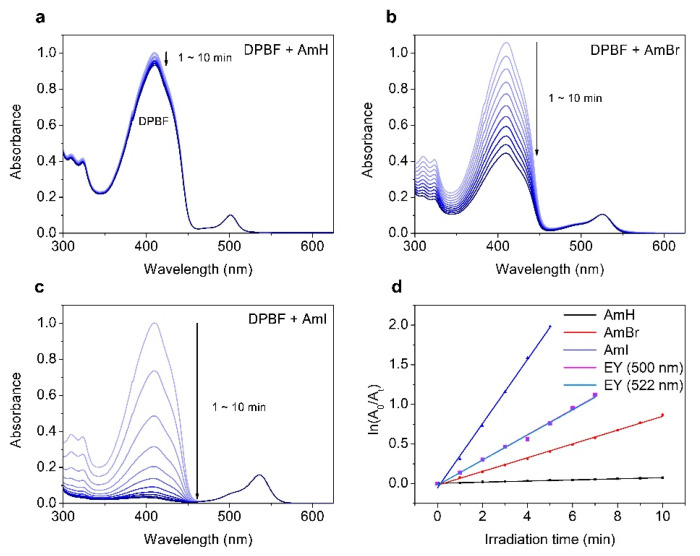
Absorption spectra of a methanol solution of DPBF measured over time with (**a**) AmH, (**b**) AmBr, and (**c**) AmI under irradiation by a green LED light (λ_max_ = 500 nm for AmH and EY; λ_max_ = 522 nm for AmBr, AmI, and EY) with 7 mW cm^−2^ radiation intensity. (**d**) Changes in the absorbance of DPBF at 410 nm vs. time based on first-order kinetics (dots) and linear fits (line) on a semilogarithmic scale. The data of EY (500 nm) and EY (522 nm) are largely overlapped with each other. Legend: EY = Eosin Y.

The ability of a photosensitizer to generate ^1^O_2_ is crucial for its efficacy as a PDT agent. However, most BODIPY-based dyes have low singlet oxygen generative power, largely because of the intramolecular singlet-triplet excited state spin-forbidden electronic transition. For the majority of these dyes, most of the absorbed light energy is retained in singlet excited states (S_n_) and dissipated as fluorescence instead of undergoing a singlet-to-triplet (ISC) transition [35]. In addition, the amount of singlet oxygen generated depends on different factors, including the lifetime of the PS triplet state, the ability of the substituents and solvent to quench ^1^O_2_, and the energy transfer efficiency from the PS triplet state to the ^3^O_2_ ground state [36,37]. Herein, the singlet oxygen quantum yield (Φ_Δ_) was assessed by monitoring the absorbance of a well-known ^1^O_2_ scavenger, 1,3-diphenylisobenzofuran (DPBF), against a reference standard Eosin Y (Φ_Δ_ = 0.26; Figure S13 in the Supplementary Information). As shown in Table 1, the halogenated BODIPY dyes had higher values than their non-halogenated counterparts. Moreover, the iodinated BODIPY **AmI** incurred higher Φ_Δ_ (0.55) than its brominated analog **AmBr** (0.13), indicating a more pronounced heavy atom effect induced by the heavier iodine atoms. The heavy-atom effect is further demonstrated in Figure 3a–d, which shows the time-dependent absorption density decay curves and linearly fitted degradation rates for DPBF with BODIPY dyes and standard Eosin Y, respectively. After incubation with the halogenated BODIPYs **AmBr** and **AmI** and exposure to LED radiation, DPBF exhibited extensive bleaching. The distinctive absorbance band of DPBF at approximately 424 nm disappeared completely after 10 min of LED exposure. Again, the absorbance band disappeared more abruptly in BODIPY **AmI** than in BODIPY **AmBr**, which was attributed to the heavy-atom effect of iodine atoms. In contrast, the **AmH** control showed no significant DPBF photobleaching capability under identical experimental conditions.

Moreover, theoretical calculations were done to further rationalize the dynamics of the intersystem crossing of BODIPY dyes. It is imperative that the triplet states are generated prior to ^1^O_2_ production. Herein, the Φ_Δ_ is correlated with the rate of ISC (*k*_ISC_). The rate of ISC is represented by the following equation [38]:

(3) $$k_{ISC}\propto \frac{\left\langle T_{m}|H_{SO}|S_{n}^{2}\right\rangle }{{\left(∆E_{S_{n}-T_{m}}\right)}^{2}}$$

where *H*_SO_ is the Hamiltonian for the spin-orbit coupling (SOC), and Δ*E*S_n_ − T_m_ represents the energy difference between the singlet (S_n_) and triplet (T_m_) states, respectively. As shown in Equation (1), two parameters are critical to *k*_ISC_: the SOC matrix element and the Δ*E*S_n_ − T_m_ involved in the ISC, which have directly and inversely proportional relationships to *k*_ISC_, respectively. Herein, the optimized molecular geometries of the excited states were used to calculate their energies and the SOC matrix elements for a manifold of S_n_ − T_m_ states. As noted by Kasha’s rule [39], the ISC transition from the higher S_n_ (n > 1) to the triplet states is not likely to be a result of rapid internal conversion; hence, the S1 state was selected for singlet state computations. Plausible photophysical decay pathways and their corresponding transition energies are summarized in Table 2. S_1_ − T_1_ and S_1_ − T_3_ manifolds have greater SOC values than those of the S_1_ − T_2_ manifold for all compounds. Nevertheless, the large energy gaps (>0.65 eV) between these states (∆*E*_S1−T1_ and ∆*E*_S1−T3_) deter ISC transitions between S_1_ and T_m_ (m = 1, 3) states. The T_2_ state has shown energy closest to the S_1_ state, suggesting that the S_1_ − T_2_ manifold is the most plausible pathway for the ISC transition of cationic BODIPY dyes.

Moreover, the magnitudes of ∆E were comparable across all compounds (0.00–0.07 eV), showing that energy difference has a negligible effect on ISC kinetics. In contrast, the SOC parameters for the S_1_ − T_2_ manifold significantly varied. For instance, BODIPY **AmH** showed SOC values of 0.04 cm^−1^, whereas halogenated BODIPY **AmBr** and **AmI** exhibited greater SOC values. These values confirm the heavy-atom effect via spin-orbit perturbation induced by halogen atoms, particularly iodine atoms, incorporated into the BODIPY dyes. The results presented herein reinforce those reported in the literature [40,41].

### 3.4. Confocal Imaging and Photodynamic Activity of the Cationic BODIPY Dyes

**Figure 4:**
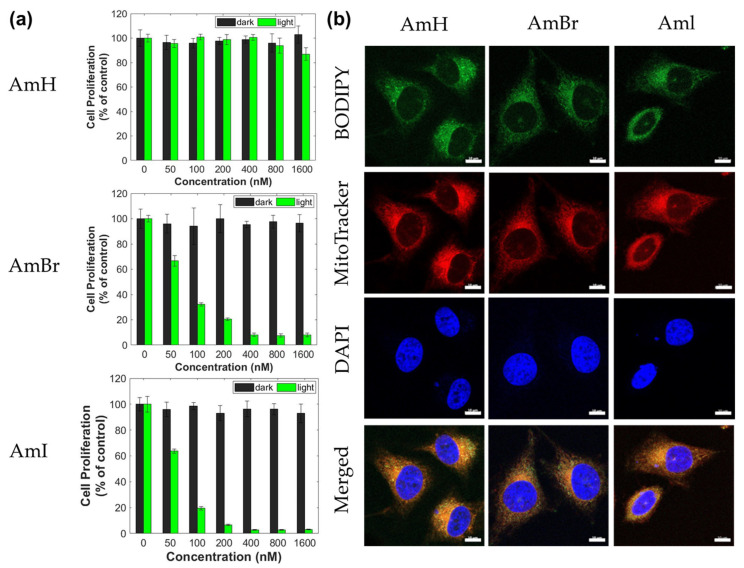
(**a**) Cell proliferation (% of control) of HeLa cancer cell lines under dark and light conditions; (**b**) CLSM images of HeLa cell line after 24 h of incubation with the BODIPY dyes (1.6 µM) with MitoTracker Red and DAPI as co-stain.

When assessing the prospect of a PDT agent for therapeutic use, cellular viability and cytotoxicity are important factors to consider. In particular, PS exhibited negligible cytotoxicity in the dark and notable toxicity when exposed to light. In this study, the cytotoxicity of cationic BODIPY dyes was evaluated in HeLa and MCF-7 cancer cell lines. The colorimetric-based MTS assay was used to assess cell proliferation. This well-established protocol for assessing cellular viability relies heavily on the reduction of MTS to colored formazan in metabolically viable cells [42]. Cancer cells were initially incubated with BODIPY-containing solutions (0–1600 nM) under normal conditions to evaluate their cytotoxicity. As shown in Figure 4a, all synthesized cationic BODIPY dyes were observed to have negligible toxicity against HeLa cells in the dark, and the cells maintained >90% viability even at the highest dosage of 1600 nM. Similarly, MCF-7 cancer cells incubated with BODIPY dyes showed low cytotoxicity in the dark (Figure S14a in the Supplementary Information). All three BODIPY dyes showed 90% or higher cell viability, even after incubation at the highest concentration (1600 nM). Moreover, non-halogenated BODIPY AmH showed no significant cytotoxic effect on cancer cells, as indicated by its extremely high cell viability, even with light activation. The minimal toxicities demonstrated by the synthesized PS agents under dark conditions, even with the light illumination of AmH, are a potential indication of their excellent biocompatibility.

In contrast, cancer cell viability drastically decreased when incubated with halogenated BODIPY **AmBr** and **AmI** and subsequently irradiated with LED at 520 nm for 10 min. As illustrated in Figure 4a, the halogenated BODIPY dyes **AmBr** and **AmI** induced significant toxic effects (*p* < 0.05) on HeLa cells, even at the lowest dosage (50 nM). Also, the degree of produced cytotoxicity intensified as their concentrations increased. For MCF-7 cells, **AmBr** and **AmI** induced similar dose-dependent cytotoxicities. MCF-7 cell viability started decreasing after incubation with 50 nM BODIPY dye, and cytotoxicity increased at higher concentrations (Figure S14a). Table 3 summarizes the IC_50_ values for halogenated BODIPYs. The calculated IC_50_ values revealed that both compounds had similar phototoxic effects. The considerably lower IC_50_ for **AmI** compared to **AmBr** can be attributed to the higher production of highly toxic ^1^O_2_. These PDT results correspond with the fluorescence and singlet oxygen quantum yield measurements of the dyes, as reported in Table 1. Notably, the IC_50_ values herein are comparable to those of similarly halogenated BODIPYs but without the cationic group at the *meso* position previously reported by Gorbe et al. [25].

Morphological analysis of the active mitochondria was also performed using fluorescent bioimaging to show the cell imaging abilities of the synthesized cationic BODIPY dyes using HeLa and MCF-7 cells. After treatment with cationic BODIPY dyes, the fluorescence of cancer cell lines was examined and captured using confocal laser scanning microscopy (CLSM). All the synthesized BODIPY dyes readily entered HeLa and MCF-7 cancer cells and generated intense green fluorescence in both cells, as shown in Figure 4b and Figure S14b (Supplementary Information), respectively, demonstrating the excellent bioimaging capabilities of the synthesized dyes. Moreover, MitoTracker Red and DAPI easily entered the cancer cells and produced clear bioimages. According to the merged images, the intracellular emission by the mitochondria tracker overlapped with that of the synthesized BODIPY dyes. Interestingly, green fluorescent images can be observed in the cancer cells treated with **AmBr** and **AmI** in spite of the significant heavy-atom effect brought about by the two halogen atoms. The symmetric orientation of the halogen atoms in the halogenated BODIPY dyes neutralizes the halogenation-induced charge transfer character to bring back the initial S1-state property of the parent BODIPY H1, hence, leading to a fluorescence that was more efficient than predicted. Efficient fluorescence imaging of halogenated BODIPYs has been described in our previous publications [30,31].

### 3.5. Mitochondria-Targeting Ability of the Cationic BODIPY Derivatives

**Figure 5:**
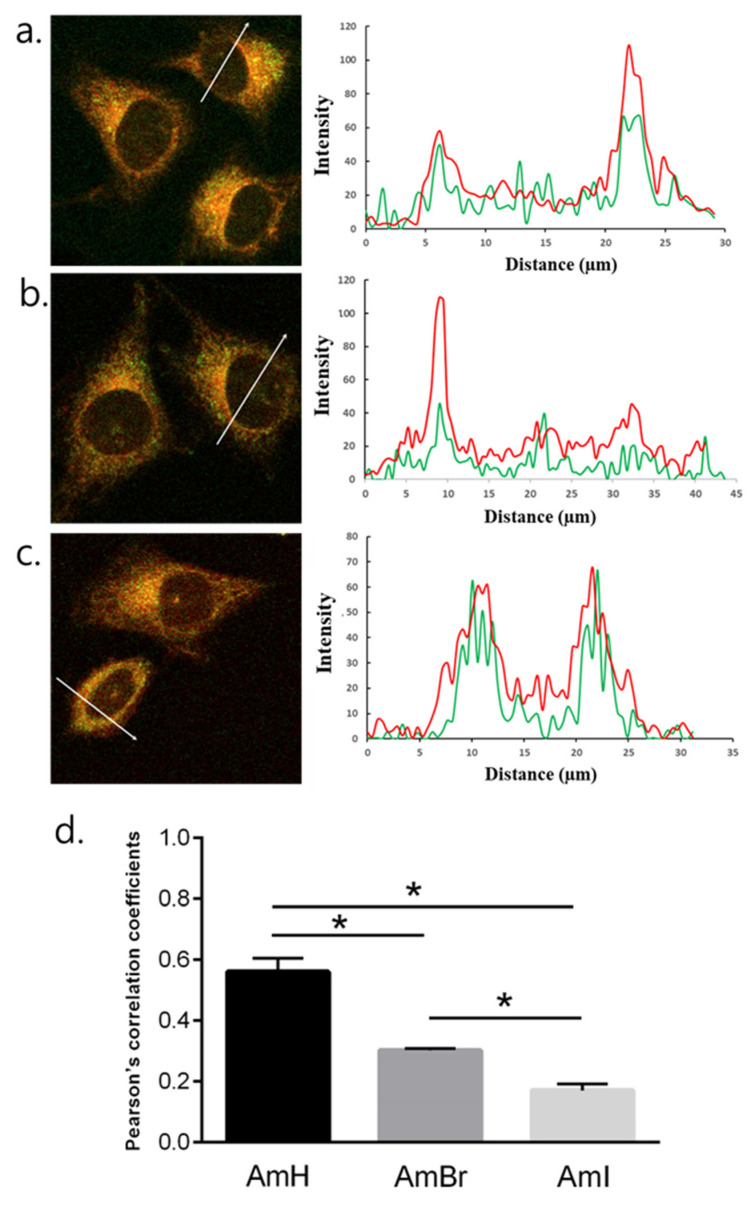
Fluorescence micrographs of HeLa cells co-stained with MitoTracker Red and their corresponding fluorescence intensity profiles along the region of interest marked by a white arrow for BODIPYs (**a**) **AmH**, (**b**) **AmBr**, and (**c**) **AmI**. The green topographic profile corresponds to each BODIPY dye, whereas the red one is for MitoTracker Red. (**d**) Pearson’s correlation coefficient of the colocalization of each BODIPY dye with mitochondria. Significant differences between group values were statistically analyzed (*, *p* < 0.05).

Intracellular colocalization analyses were conducted to evaluate the mitochondria-targeting ability of the synthesized BODIPY derivatives by co-staining cancer cells with cationic BODIPY derivatives and MitoTracker Red which is an established mitochondria-targeting dye. The fluorescence of BODIPY dyes and MitoTracker Red was observed in the green and red channels, respectively. Cancer cells were stained with DAPI, a known cell nucleus-targeting fluorescence marker, as a negative control. Herein, a profiling of the fluorescence intensity line emitted by BODIPY dyes and MitoTracker Red in the mitochondria was analyzed in detail. The fluorescent signals from the BODIPY dyes in HeLa cells corresponded to those of MitoTracker Red, as shown in Figure 5, demonstrating the ability of the synthesized cationic BODIPY dyes to selectively target and localize to mitochondria. Furthermore, the Pearson correlation coefficients calculated based on the fluorescent signals of BODIPY and tracker dye for AmH, AmBr, and AmI are 0.57, 0.30 and 0.18, respectively (Figure 5d). The same fluorescence intensity profiles were observed for the colocalization of BODIPY dyes in MCF-7 cells (Figure S15 in the Supplementary Materials). These findings are in good agreement with previously published literature on cation-containing fluorophores that have dual functionality in PDT and fluorescence imaging [43,44]. The ability of PSs to target cancer cells stems from the fact that their membrane potential is higher in cancer cells than in normal cells [45]. This large gap in the mitochondrial membrane potential accelerates the accumulation of cationic lipophilic compounds, increasing their concentration in the mitochondria by up to 500 times [21]. Overall, the results show that the BODIPY derivatives are feasible compounds to act as excellent agents for fluorescence bioimaging and as effective PSs that significantly produce triggerable toxicity for the photodynamic treatment of cancer.

## 4. Conclusions

We synthesized a series of cationic BODIPY dyes and examined their photophysical, mitochondria-targeting, and photodynamic therapeutic capabilities as photosensitizers for cancer therapy. Ammonium-functionalized BODIPY derivatives are water soluble and can generate highly toxic singlet oxygen species. Owing to the heavy-atom effect, the singlet oxygen quantum yields of the halogenated BODIPY dyes, **AmBr** and **AmI**, were significantly high (0.13 and 0.55, respectively). Moreover, there was no significant cytotoxicity of the BODIPY dyes after incubating the subject cancer cell lines in the dark. In contrast, halogenated BODIPYs showed superior and dose-dependent cytotoxicity upon irradiation of all cancer cells under investigation while also displaying excellent mitochondrial-targeting ability, demonstrating their suitability as photosensitizers for photodynamic cancer therapy. Overall, the results described herein present an effective strategy to synthesize mitochondria-targeting BODIPY dyes with PDT potential and provide insight into the development and application of these dyes as more efficient BODIPY-based PDT photosensitizers.

## Figures and Tables

**Table 1:** Photophysical and photosensitizing properties of cationic BODIPY dyes. ^a,b^ Wavelengths of maximum absorption and emission; ^c^ in ethanolic solution; ^d^ in methanolic solution.

Sample	λ_abs_ (nm) ^a^	λ_emi_ (nm) ^b^	Φ_F_^c^	Φ_Δ_^d^
AmH	501	513	0.34	0.01
AmBr	526	546	0.06	0.13
AmI	536	558	0.01	0.55

**Table 2:** Singlet and triplet electronic transition energies (*E*), energy difference (∆*E*), and spin-orbit coupling (SOC) matrix element for S_1_ − T_m_ (m = 1, 2, 3) manifold.

	Manifold	$E_{S_{1}}(eV)$ ^a^	$E_{T_{m}}(eV)$ ^a^	$∆E_{S_{1}-T_{m}}(eV)$ ^a^	$SOC({cm}^{-1})$ ^b^
**AmH**	S_1_ − T_1_		1.52	1.40	0.74
	S_1_ − T_2_	2.92	2.99	0.07	0.04
	S_1_ − T_3_		4.53	1.61	0.14
**AmBr**	S_1_ − T_1_		1.52	1.19	0.86
	S_1_ − T_2_	2.71	2.71	0.00	0.40
	S_1_ − T_3_		4.08	1.37	0.16
**AmI**	S_1_ − T_1_		1.56	1.10	3.33
	S_1_ − T_2_	2.66	2.67	0.01	0.99
	S_1_ − T_3_		3.95	1.29	0.44

^a^ Computed using Gaussian 16 at the B3LYP/6-31G(d) level of theory for **AmH** and **AmBr**. Computed using Gaussian 16 at the B3LYP/LANL2DZ level of theory for **AmI**. ^b^ Computed at the B3LYP/ZORA-def2-TZVP level of theory using the ORCA 5.0.2 program.

**Table 3:** Calculated IC_50_ of AmBr and AmI dyes in HeLa and MCF-7 cell lines in nM.

	HeLa	MCF-7
AmBr	64.54 ± 3.00	48.57 ± 3.51
AmI	59.27 ± 0.53	49.35 ± 0.52

## Data Availability

The data presented in this study are available on request from the corresponding authors.

## Supplementary Materials

The following supporting information can be downloaded at: https://www.mdpi.com/article/10.3390/pharmaceutics15051512/s1. Figure S1. ^1^H-NMR spectrum of H1. Figure S2. ^1^H-NMR spectrum of Br1. Figure S3. ^1^H-NMR spectrum of I1. Figure S4. ^1^H-NMR spectrum of cationic BODIPY AmH. Figure S5. ^13^C-NMR spectrum of cationic BODIPY AmH. Figure S6. HR-ESI mass spectrum of cationic BODIPY AmH. Figure S7. ^1^H-NMR spectrum of cationic BODIPY AmBr. Figure S8. ^13^C-NMR spectrum of cationic BODIPY AmBr. Figure S9. HR-ESI mass spectrum of cationic BODIPY AmBr. Figure S10. ^1^H-NMR spectrum of cationic BODIPY AmI. Figure S11. ^13^C-NMR spectrum of cationic BODIPY AmI. Figure S12. HR-ESI mass spectrum of cationic BODIPY AmI. Table S1. Transition energy (E), wavelength (λ), and oscillator strength (f) for the lowest singlet excited state of BODIPY PSs and the contribution of frontier orbitals to each transition. Figure S13. (a,b) Time-dependent absorption spectra of air-saturated methanol solution of DPBF containing EY under green LED light irradiation (500 nm and 522 nm, 7 mW cm^−2^). (c,d) Temporal change in the absorbance of DPBF at 410 nm plotted according to the first order kinetics (dots) with linear fits (line) in semilogarithmic scale. Legend: EY: Eosin Y. Figure S14. (a) Cell proliferation (% of control) of MCF-7 cancer cell lines under dark and light conditions; (b) CLSM images of MCF-7 cell line after a 24-h incubation with the BODIPY dyes (1.6 μM) with MitoTracker Red and DAPI as co-stain. Figure S15. Fluorescence micrographs of MCF-7 cells co-stained with MitoTracker Red and their corresponding fluorescence intensity profiles along the region of interest marked by a white arrow for BODIPYs (a) AmH, (b) AmBr, and (c) AmI. The green topographic profile corresponds to each BODIPY dye while the red one is for MitoTracker Red.
