# Supplementary material for: Cationic BODIPY Photosensitizers for Mitochondrion-Targeted Fluorescence Cell-Imaging and Photodynamic Therapy
Source: Pharmaceutics. 2023 May 16;15(5):1512. doi: 10.3390/pharmaceutics15051512 (PMC10223002; doi:10.3390/pharmaceutics15051512)
Supplement: Supplementary file 1 [file pharmaceutics-15-01512-s001.zip › pharmaceutics-2383326-supplementary.pdf]

Supplementary Information

Cationic BODIPY Photosensitizers for Mitochondrion-Targeted Fluorescence Cell-Imaging and Photodynamic Therapy

Isabel Wen Badon [1,2,†], Jun-Pil Jee [3,†], Temmy Pegarro Vales [4,5], Chanwoo Kim [6], Seungbin Lee [6], Jaesung Yang [6,*], Si Kyung Yang [7,*] and Ho-Joong Kim [1,*]

1 Department of Chemistry, Chosun University, Gwangju 61452, Republic of Korea; istbadon@gmail.com
2 Department of Life Sciences, Chung-Ang University, Seoul 06974, Republic of Korea
3 Drug Delivery Research Lab, College of Pharmacy, Chosun University, Gwangju 61452, Republic of Korea; jee@chosun.ac.kr
4 Department of Chemistry, Caraga State University, Butuan City 8600, Philippines; valestemmy@gmail.com
5 Mineral Resources Management Research and Training Center, Caraga State University, Butuan City 8600, Philippines
6 Department of Chemistry, Yonsei University, Wonju 26493, Republic of Korea; chanwoo.kim@yonsei.ac.kr (C.K.); dltmdqls0503@gmail.com (S.L.)
7 Department of Chemistry Education, Chonnam National University, Gwangju 61186, Republic of Korea
* Correspondence: jaesung.yang@yonsei.ac.kr (J.Y.); sky223@jnu.ac.kr (S.K.Y.); hjkim@chosun.ac.kr (H.-J.K.)
† These authors contributed equally to this work.

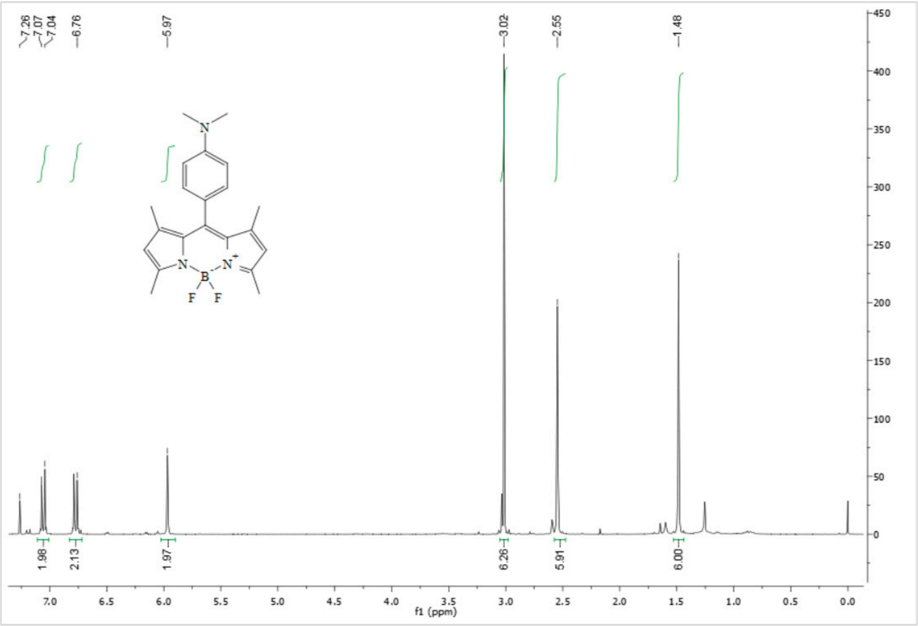

Figure S1. ^{1}H-NMR spectrum of H1.

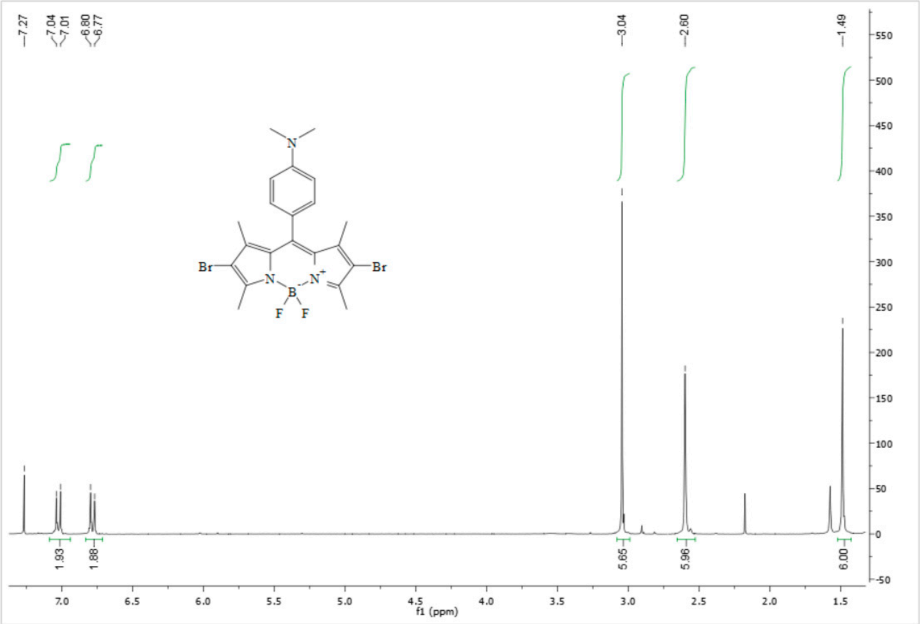

Figure S2. ^{1}H-NMR spectrum of Br1.

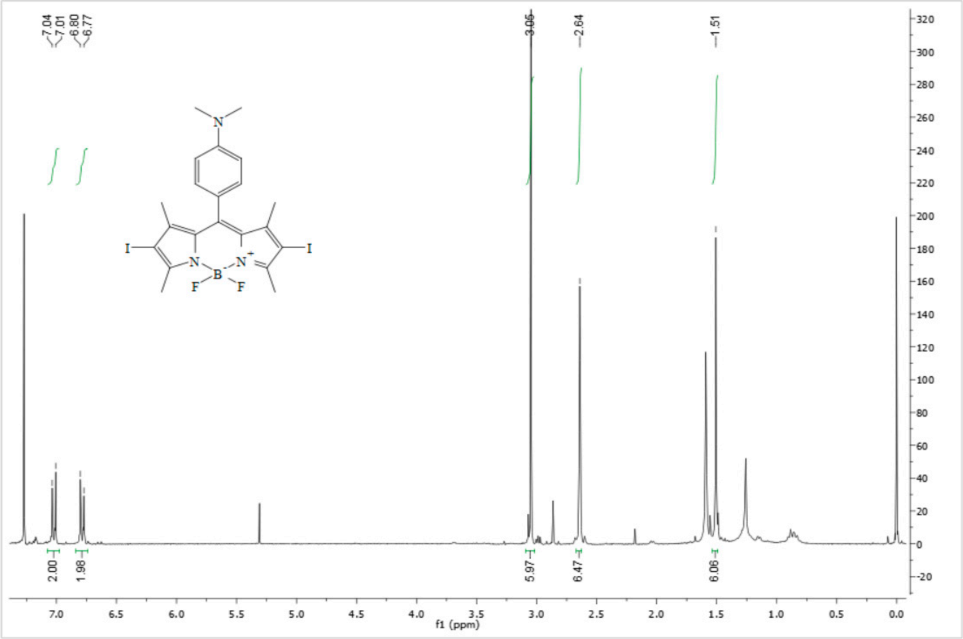

Figure S3. ^{1}H-NMR spectrum of I1.

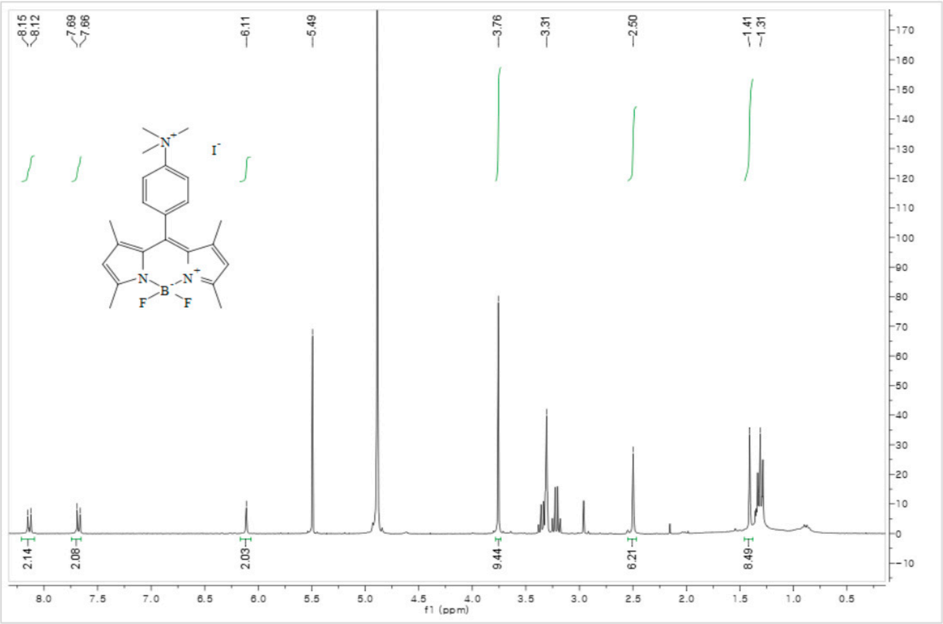

Figure S4. ^{1}H-NMR spectrum of cationic BODIPY AmH.

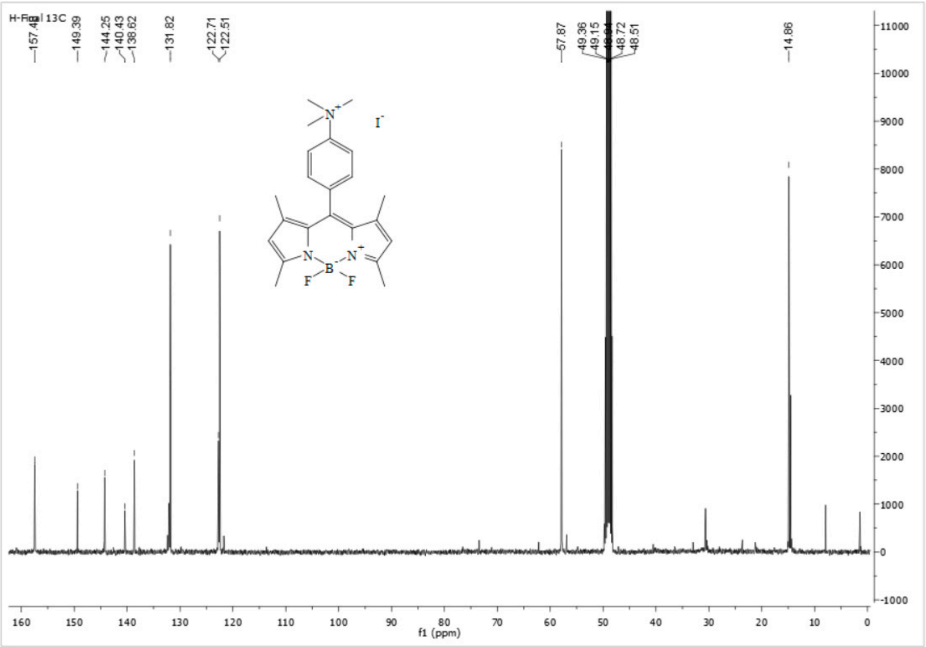

Figure S5. ^{13}C-NMR spectrum of cationic BODIPY AmH.

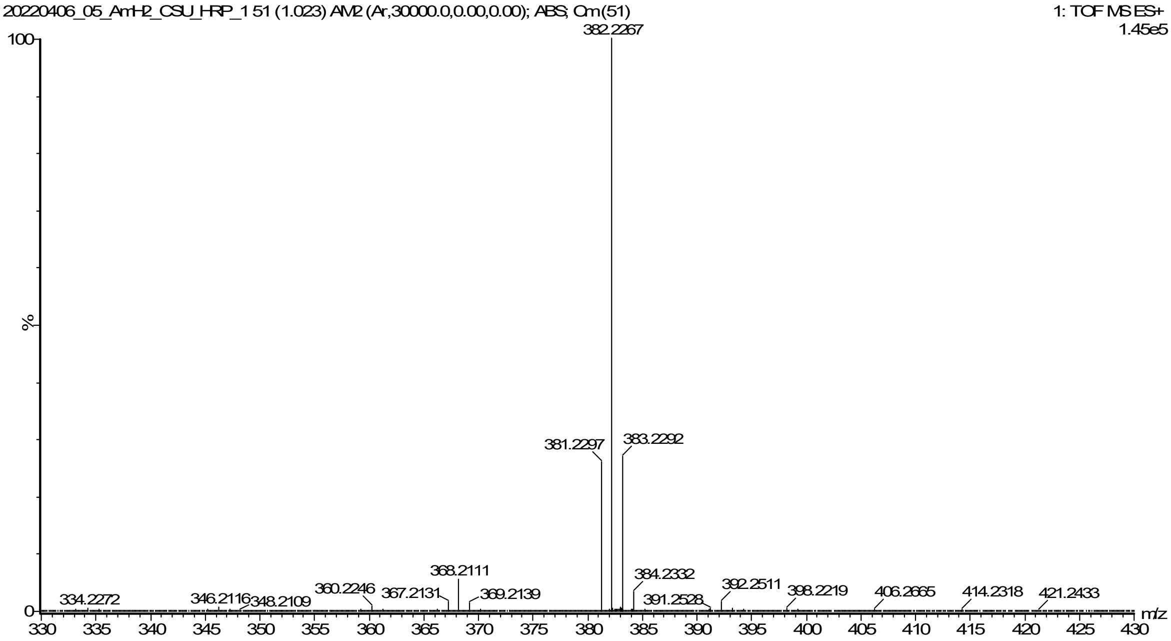

Figure S6. HR-ESI mass spectrum of cationic BODIPY AmH.

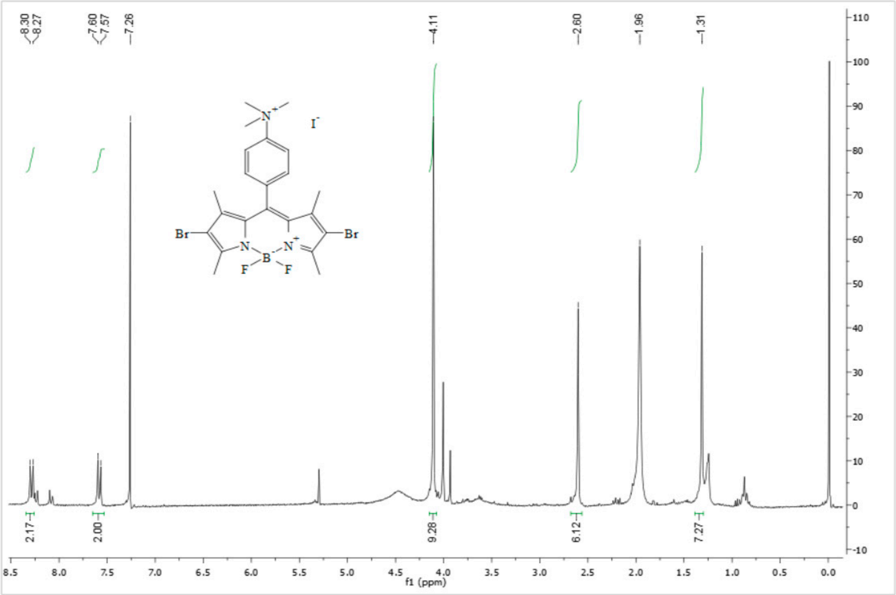

Figure S7. ^{1}H-NMR spectrum of cationic BODIPY AmBr.

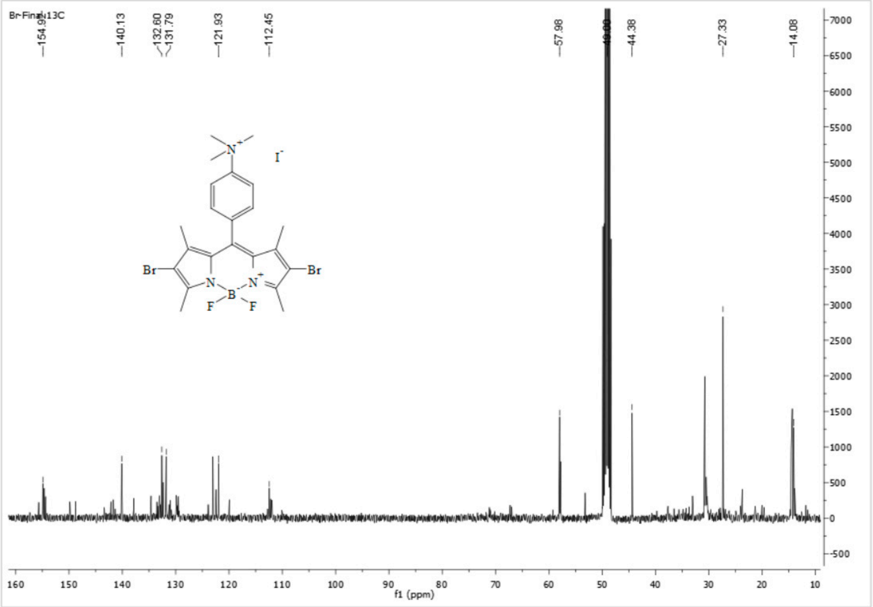

Figure S8. ^{13}C-NMR spectrum of cationic BODIPY AmBr.

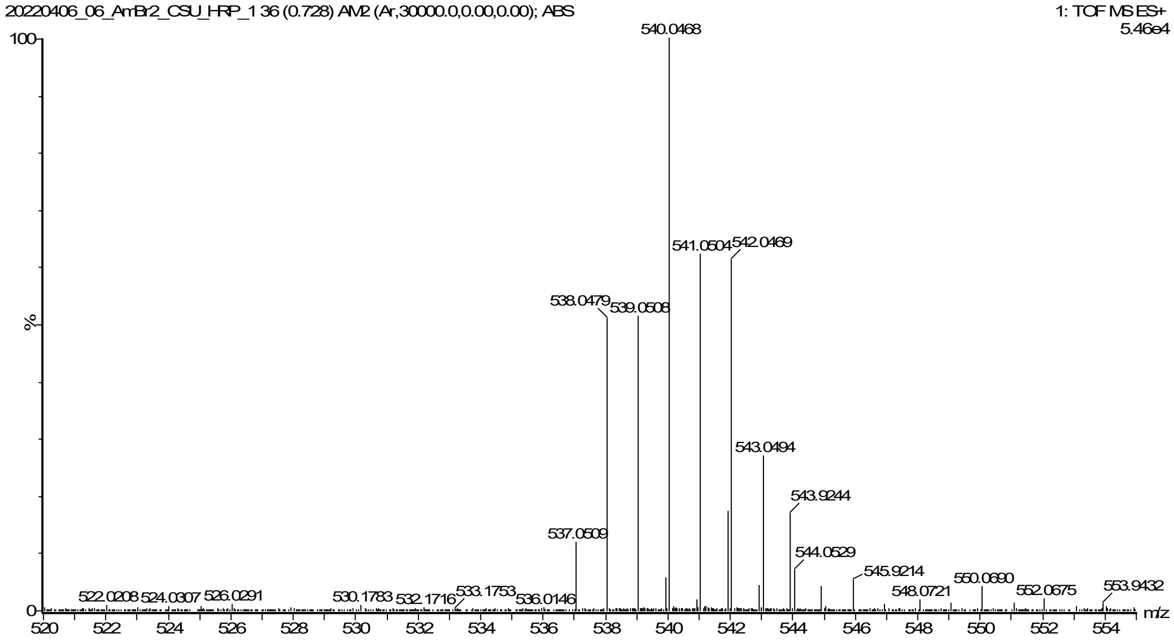

Figure S9. HR-ESI mass spectrum of cationic BODIPY AmBr.

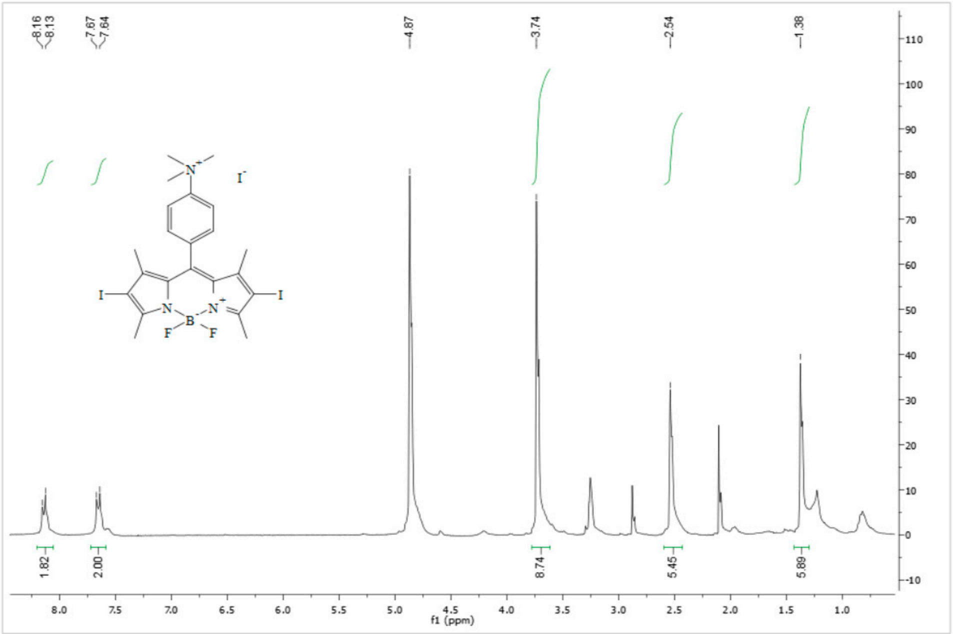

Figure S10. ^{1}H-NMR spectrum of cationic BODIPY AmI.

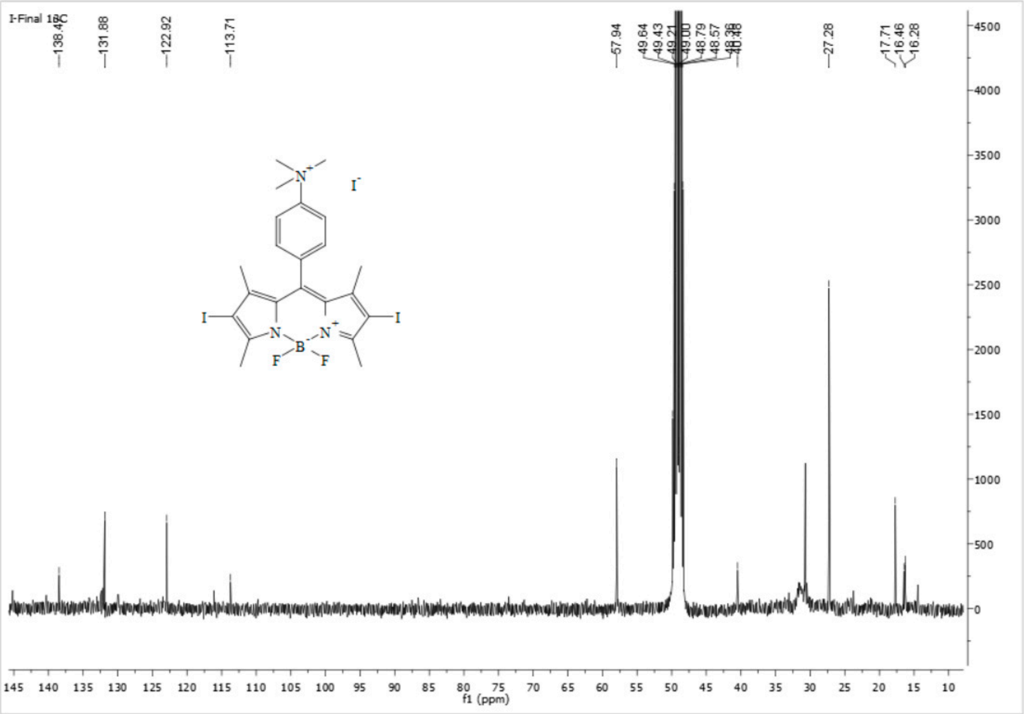

Figure S11. ^{13}C-NMR spectrum of cationic BODIPY AmI.

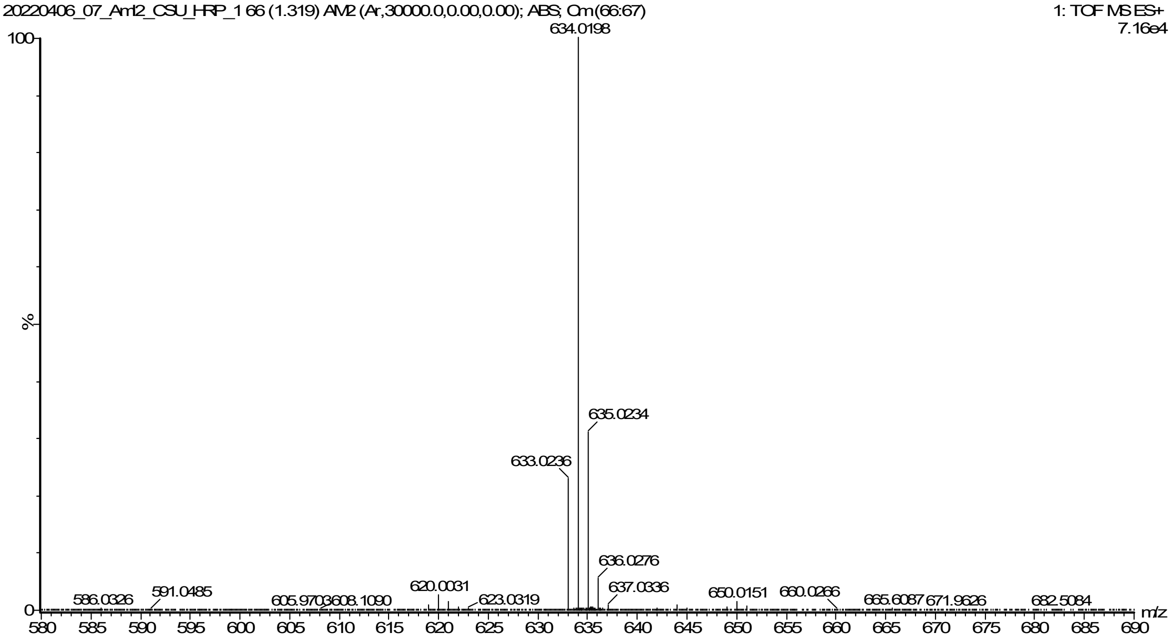

Figure S12. HR-ESI mass spectrum of cationic BODIPY AmI.

Table S1. Transition energy (E), wavelength (λ), and oscillator strength (f) for the lowest singlet excited state of BODIPY PSs and the contribution of frontier orbitals to each transition.

	State	E (eV)[a]	λ (nm)	f[b]	Major contribution to transition
AmH	S_1	2.92	425	0.5636	H → L (97%)
AmBr	S_1	2.71	458	0.5213	H → L (91%), H-1 → L (10%)
AmI	S_1	2.66	466	0.4975	H → L (88%), H-1 → L (12%)

[a] Vertical excitation energy computed in water using B3LYP/6-31g(d) for AmH and AmBr. Vertical excitation energy computed in water using B3LYP/LANL2DZ for AmI. [b] Oscillator strength.

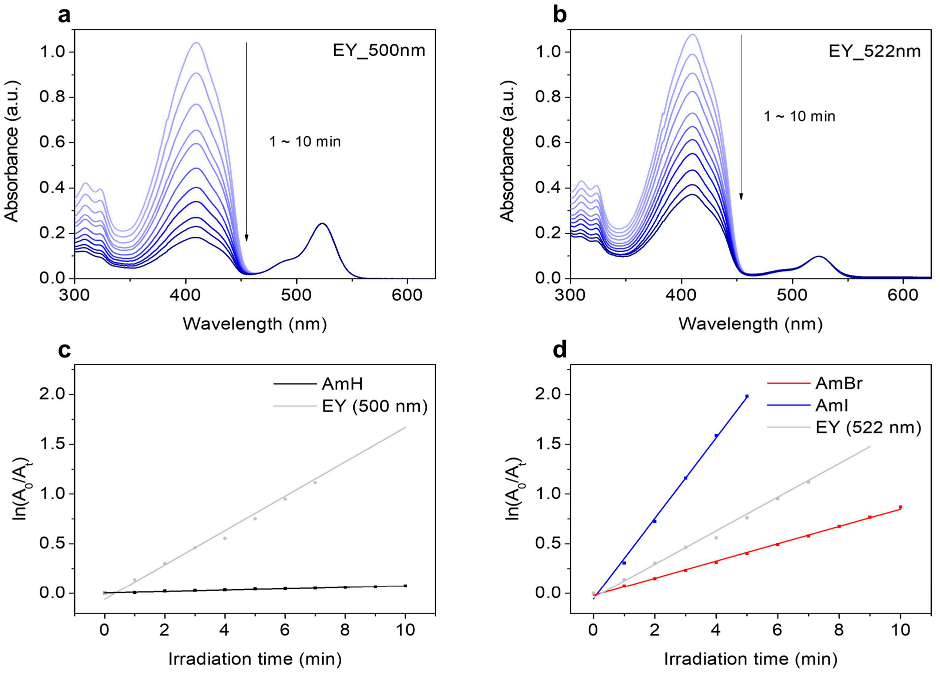

Figure S13. (a–b) Time-dependent absorption spectra of air-saturated methanol solution of DPBF containing EY under green LED light irradiation (500 nm and 522 nm, 7 mW cm^{-2}). (c-d) Temporal change in the absorbance of DPBF at 410 nm plotted according to the first order kinetics (dots) with linear fits (line) in semilogarithmic scale. Legend: EY : Eosin Y.

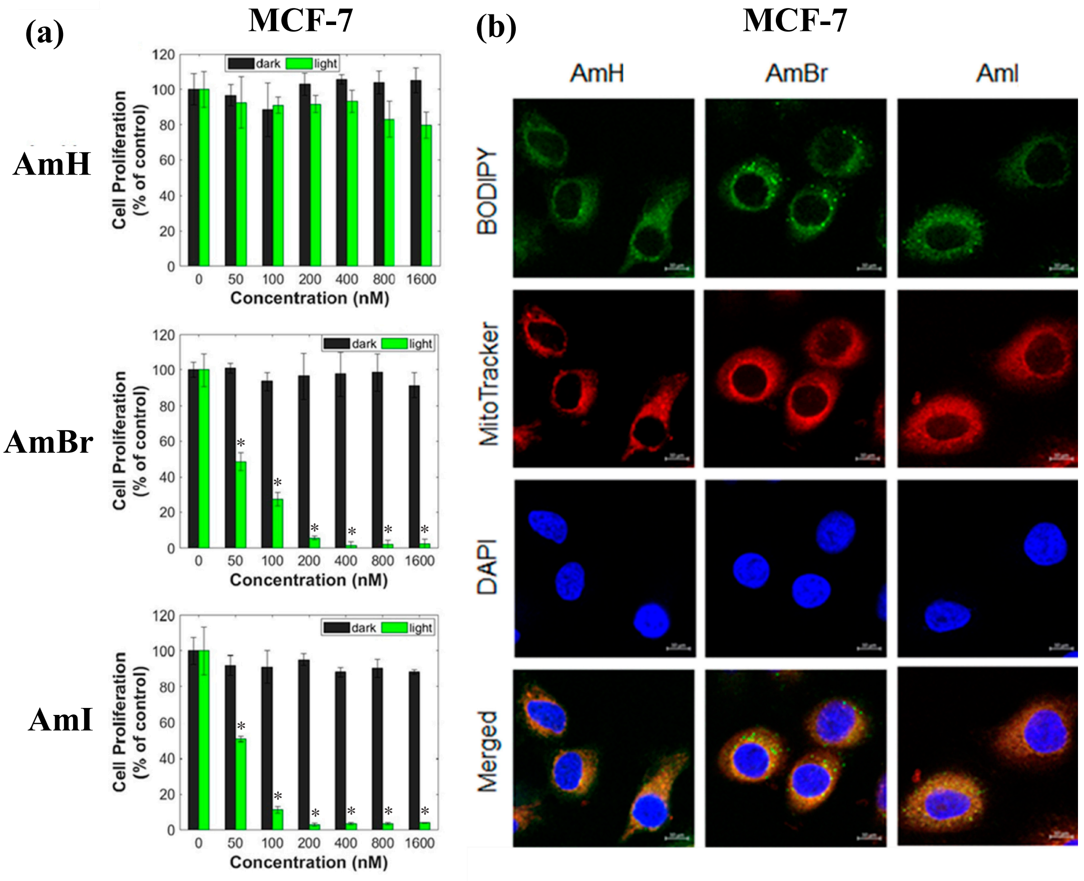

Figure S14. (a) Cell proliferation (% of control) of MCF-7 cancer cell lines under dark and light conditions; (b) CLSM images of MCF-7 cell line after a 24-hour incubation with the BODIPY dyes (1.6 μM) with MitoTracker Red and DAPI as co-stain.

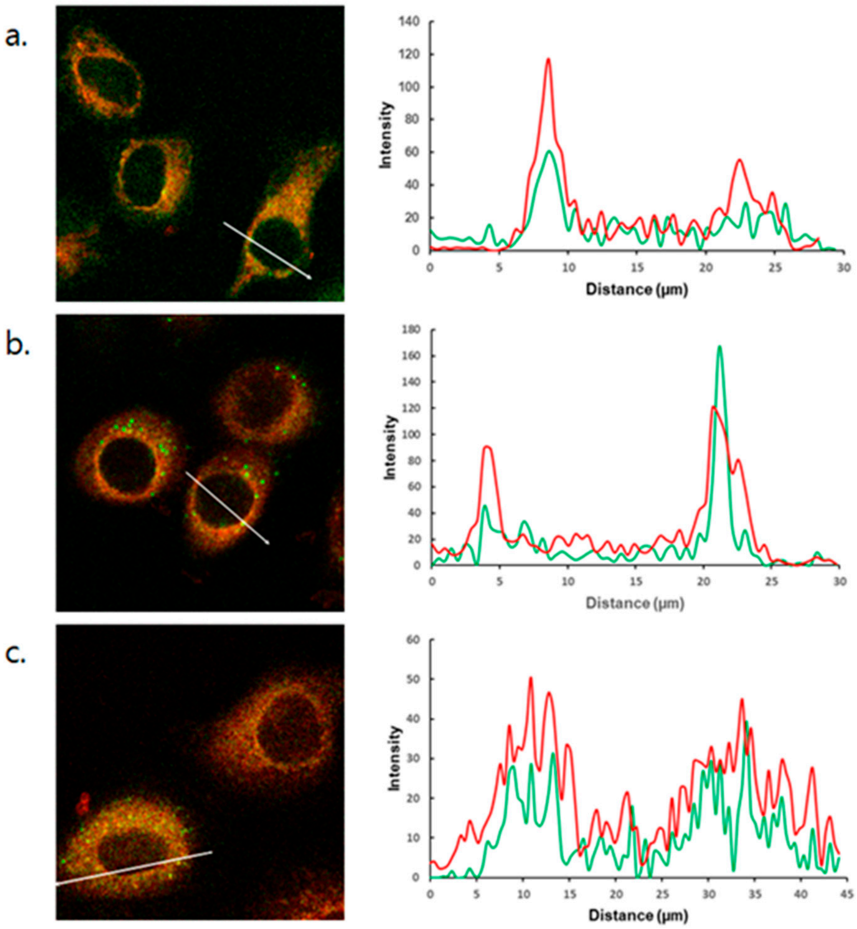

Figure S15. Fluorescence micrographs of MCF-7 cells co-stained with MitoTracker Red and their corresponding fluorescence intensity profiles along the region of interest marked by a white arrow for BODIPYs (a) AmH, (b) AmBr, and (c) AmI. The green topographic profile corresponds to each BODIPY dye while the red one is for MitoTracker Red.
